# *Vital Signs*: Suicide Rates and Selected County-Level Factors — United States, 2022

**DOI:** 10.15585/mmwr.mm7337e1

**Published:** 2024-09-19

**Authors:** Alison L. Cammack, Mark R. Stevens, Rebecca B. Naumann, Jing Wang, Wojciech Kaczkowski, Jorge Valderrama, Deborah M. Stone, Robin Lee

**Affiliations:** ^1^Division of Injury Prevention, National Center for Injury Prevention and Control, CDC; ^2^Guidehouse, McLean, Virginia.

## Abstract

**Introduction:**

Approximately 49,000 persons died by suicide in the United States in 2022, and provisional data indicate that a similar number died by suicide in 2023. A comprehensive approach that addresses upstream community risk and protective factors is an important component of suicide prevention. A better understanding of the role of these factors is needed, particularly among disproportionately affected populations.

**Methods:**

Suicide deaths were identified in the 2022 National Vital Statistics System. County-level factors, identified from federal data sources, included health insurance coverage, household broadband Internet access, and household income. Rates and levels of factors categorized by tertiles were calculated and presented by race and ethnicity, sex, age, and urbanicity.

**Results:**

In 2022, the overall suicide rate was 14.2 per 100,000 population; rates were highest among non-Hispanic American Indian or Alaska Native (AI/AN) persons (27.1), males (23.0), and rural residents (20.0). On average, suicide rates were lowest in counties in the top one third of percentage of persons or households with health insurance coverage (13.0), access to broadband Internet (13.3), and income >100% of the federal poverty level (13.5). These factors were more strongly associated with lower suicide rates in some disproportionately affected populations; among AI/AN persons, suicide rates in counties in the highest tertile of these factors were approximately one half the rates of counties in the lowest tertile.

**Conclusions and Implications for Public Health Practice:**

Higher levels of health insurance coverage, household broadband Internet access, and household income in communities might play a role in reducing suicide rates. Upstream programs, practices, and policies detailed in CDC’s Suicide Prevention Resource for Action can be implemented by decision-makers, government agencies, and communities as they work together to address community-specific needs and save lives.

**Summary**

**What is already known about this topic?**

In 2022, approximately 49,000 persons died by suicide in the United States. A comprehensive approach that addresses health-related community factors, such as health care access, social and community context, and economic stability, could help prevent suicide.

**What is added by this report?**

Suicide rates were lowest in counties with the highest health insurance coverage, broadband Internet access, and income. These factors were more strongly associated with lower suicide rates in some groups that are disproportionately affected by suicide.

**What are the implications for public health practice?**

Implementing programs, practices, and policies that improve the conditions in which persons are born, grow, live, work, and age might be an important component of suicide prevention efforts. Decision-makers, government agencies, and communities can work together to address community-specific needs and save lives.

## Introduction

In 2022, approximately 49,000 persons died by suicide in the United States (age-adjusted suicide rate = 14.2 per 100,000 population), and provisional data indicate a similar number of persons died by suicide in 2023 (*1*). Suicide was the second leading cause of death among persons aged 10–34 years in 2022 (*1*). Several demographic groups are disproportionately affected by suicide in the United States (*2*). These groups include males, rural residents, and persons from certain racial and ethnic groups, particularly non-Hispanic American Indian or Alaska Native (AI/AN) persons (*1*).

Suicide rates have increased during the last 20 years and remain high (*1*): on average one person dies by suicide every 11 minutes (*1*). However, despite these concerning data, suicide is a preventable public health problem. Suicide prevention requires a comprehensive public health approach that addresses multiple modifiable suicide risk and protective factors at the individual, relationship, community, and societal levels (*3*). Such an approach includes implementation of upstream policies, programs, and practices to prevent persons from reaching a crisis point, and downstream prevention focused on treatment, crisis intervention, and postvention (i.e., activities that reduce risk and promote healing in suicide loss survivors after a suicide has taken place).

A number of nonmedical factors that affect health outcomes, often described as social determinants of health, play an important role in shaping upstream suicide prevention efforts (*4*). These factors are the conditions in which persons are born, grow, work, live, and age.* For example, insurance coverage, access to broadband Internet, and higher household income might decrease suicide risk by improving health care access, increasing job opportunities, and providing access to sources of support and information (*5*–*7*). However, although evidence of associations between higher levels of these factors and reduced suicide risk exists (*5*–*7*), this evidence is more limited among groups disproportionately affected by suicide. To guide opportunities for prevention, CDC examined differences in suicide rates according to three specific county-level factors, overall and within demographic groups: 1) health insurance coverage, 2) broadband Internet access, and 3) income.

## Methods

### Ascertainment of Suicide Deaths

Suicide deaths from the 2022 National Vital Statistics System (NVSS) mortality files were identified using the *International Classification of Diseases, Tenth Revision* underlying cause of death codes X60–X84, Y87.0, and U03.^†^^,^^§^ Demographic factors were extracted, including data on decedent race and ethnicity (i.e., AI/AN, Asian and Native Hawaiian or Pacific Islander [Asian and NH/PI],^¶^ Black or African American [Black], White, Hispanic or Latino [Hispanic], and multiracial), sex, and age group (10–24,** 25–44, 45–64, and ≥65 years). Hispanic decedents could be of any race; all other racial and ethnic groups were non-Hispanic. Decedent county of residence was linked to the 2023 U.S. Department of Agriculture Rural-Urban Continuum Codes and categorized as urban or rural.^††^

### County-Level Factors

Three county-level factors (health insurance coverage, broadband Internet access, and household income) were measured and linked with decedent county of residence. These three factors were selected based on published literature and their relevance to multiple suicide prevention strategies, including those in CDC’s Suicide Prevention Resource for Action (*3*). Health insurance coverage was assessed as the percentage of persons in the county who had health insurance, measured using 2021 Small Area Health Insurance Estimates (SAHIE).^§§^ Broadband Internet access was defined as the percentage of households in the county that had a broadband Internet subscription, measured using 5-year estimates from the 2018–2022 American Community Survey.^¶¶^ Income level was derived from the percentage of persons in the county with household incomes >100% of the federal poverty level, measured using 2022 Small Area Income and Poverty Estimates.*** Counties were categorized into tertiles of each individual factor (i.e., counties with the highest, middle, and lowest third for percentage of persons or households with a factor).^†††^

### Data Analysis

Suicide rates (suicide deaths per 100,000 population) were calculated by tertiles of health insurance coverage, household broadband internet access, and household income, overall and by demographic subgroups. Rates were calculated using U.S. postcensal single race estimates of the July 1, 2022, residential population as denominators. Age-adjusted rates were calculated by the direct method,^§§§^ using the 2000 U.S. standard population. Differences (examined for each factor individually) in suicide rates between the counties in the highest and lowest tertiles for each factor and counties in the intermediate and lowest tertiles for each factor were compared using Z-tests when the number of suicide deaths was ≥100; p-values <0.05 were considered statistically significant. When the number of suicide deaths was <100, differences in rates were considered significant if CIs, based on a gamma distribution, did not overlap. Rate ratios (RRs) were also computed to quantify associations between levels of factors and suicide rates (i.e., RRs for counties in the highest versus lowest tertiles of factors and RRs for counties in the intermediate versus lowest tertiles of factors). Analyses were conducted using SAS software (version 9.4; SAS Institute) and R software (version 4.4.0; The R Foundation). This activity was reviewed by CDC, deemed not research, and was conducted consistent with applicable federal law and CDC policy.^¶¶¶^

## Results

### Suicide Deaths and Rates, Overall and by Demographic Factors

In 2022, a total of 49,476 suicides occurred in the United States (age-adjusted rate = 14.2 per 100,000 population) (Table 1). Among all racial and ethnic groups, the highest rates were among AI/AN persons (27.1), followed by White persons (17.6); approximately 75% of all suicides were among White persons (37,481). The suicide rate among males (23.0) was nearly four times that among females (5.9) and was higher among rural residents (20.0) than among urban residents (13.4). By age group, rates were highest among persons aged 25–44 (18.9) and 45–64 years (19.0).

**TABLE 1:** Suicide rates by race and ethnicity, sex, age group, and urbanicity — National Vital Statistics System, United States, 2022

Demographic group	Suicide deaths	Rate*
**Overall^†^**	**49,476**	**14.2**
**Race and ethnicity** ^†,§^		
AI/AN	650	27.1
Asian and NH/PI	1,554	7.1
Black or African American	3,826	8.9
White	37,481	17.6
Hispanic or Latino	5,122	8.1
Multiracial	682	10.5
**Sex** ^†^		
Female	10,203	5.9
Male	39,273	23.0
**Age group, yrs**^¶,^**^,††^		
10–24	6,533	10.0
25–44	16,848	18.9
45–64	15,645	19.0
≥65	10,438	18.1
**Urbanicity** ^§§,¶¶^		
Urban	40,096	13.4
Rural	9,359	20.0

**Abbreviations**: AI/AN = American Indian or Alaska Native; NH/PI = Native Hawaiian or Pacific Islander.

* Suicide deaths per 100,000 population.

^†^Age-adjusted rates, as described by https://wonder.cdc.gov/wonder/help/ucd-expanded.html#Age-Adjusted%20Rates. Hispanic or Latino (Hispanic) decedents could be of any race; all other racial and ethnic groups were non-Hispanic.

^§^ Race or ethnicity missing for 161 deaths.

^¶^ Crude rates.

** Age missing for three deaths.

^††^ Suppression of persons aged <10 years due to low death counts.

^§§^ Age-adjusted rates (calculated via direct method, using 2000 U.S. standard population) used 10 age group categories for age-adjustment: 0–4, 5–14, 15–24, 25–34, 35–44, 45–54, 55–64, 65–74, 75–84, and ≥85 years. National Vital Statistics System data was used for all states except Connecticut, where state vital records were used (data provided for 377 of 398 suicide deaths among Connecticut residents).

^¶¶^ Rural-Urban Continuum Codes 1–3 were coded as urban, and Codes 4–9 were coded as rural. https://www.ers.usda.gov/data-products/rural-urban-continuum-codes/

### Suicide Rates by County-Level Factors

**FIGURE 1:**
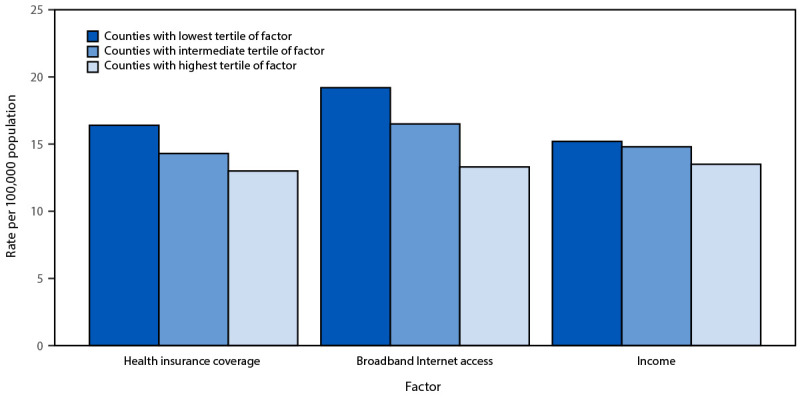
Suicide rates,* by tertiles of selected county-level factors^†^^,^^§^^,^^¶^^,^** — National Vital Statistics System,^††^ United States, 2022 **Abbreviation:** FIPS = Federal Information Processing Standard. * Age-adjusted rates (calculated via direct method, using 2000 U.S. standard population) used 10 categories for age adjustment: 0–4, 5–14, 15–24, 25–34, 35–44, 45–54, 55–64, 65–74, 75–84, and ≥85 years. ^†^ Percentage of persons with health insurance coverage. Connecticut and Valdez-Cordova Census Area, Alaska, and its updated split FIPS codes (Chugach and Copper River Census Areas, Alaska) were excluded. Data was not available for Kalawao County, Hawaii. Data for 2021 are available at https://www.census.gov/programs-surveys/sahie.html. ^§^ Percentage of households with a broadband Internet subscription. Valdez-Cordova Census Area, Alaska, and its updated split FIPS codes (Chugach and Copper River Census Areas, Alaska) were excluded. Five-year estimates (2018–2022) are available at https://www.census.gov/programs-surveys/acs. ^¶^ Percentage of persons living in a household with income >100% of the federal poverty level. Valdez-Cordova Census Area, Alaska, and its updated split FIPS codes (Chugach and Copper River Census Areas, Alaska) were excluded. Data was not available for Kalawao County, Hawaii. Data for 2022 are available at https://www.census.gov/programs-surveys/saipe.html. ** The county tertile cutoffs for the percentage of residents or households with a given factor were as follows: health insurance coverage: 53.7%–87.0%, 87.1%–91.7% and 91.7%–97.6%; broadband Internet access: 36.0%–80.6%, 80.6%–86.0%, and 86.0%–100%; and income >100% of the federal poverty level: 57.6%–83.9%, 84.0%–88.3%, and 88.4%–96.9%. Percentages were rounded to one decimal place for readability, but groups do not overlap; statistical ranking was used to split counties into tertile groups before rounding. ^††^ Data from state vital records were used for 377 of 398 suicide deaths among Connecticut residents.

Overall, average suicide rates were inversely related to each of the three county-level factor tertiles (Figure 1). Suicide rates were highest in counties in the lowest tertile of health insurance coverage (16.4), broadband Internet access (19.2), and household income (15.2), followed by counties in the intermediate tertiles (14.3, 16.5, and 14.8, respectively). The lowest suicide rates occurred in counties in the highest tertiles (13.0, 13.3, and 13.5, respectively). These findings correspond to 26%, 44%, and 13% lower suicide rates in counties in the highest versus lowest tertiles of health insurance coverage, broadband Internet access, and household income, respectively.****

### Suicide Rates and RRs by County-Level Factors and Demographic Groups

**FIGURE 2:**
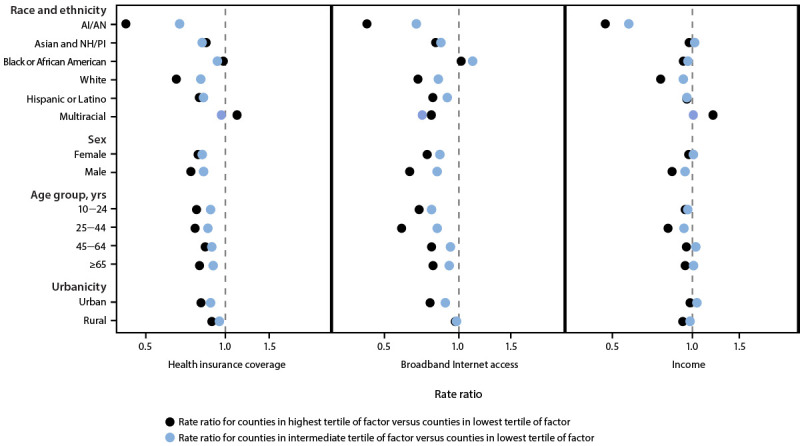
Associations between selected county-level factors*^,^^†^^,^^§^^,^^¶^ and suicide rates,** by demographic group^††^^,^^§§^^,^^¶¶^ — National Vital Statistics System,*** United States, 2022^†††^ **Abbreviations: **AI/AN = American Indian or Alaska Native; FIPS = Federal Information Processing Standard; NH/PI = Native Hawaiian or Pacific Islander. * Percentage of persons with health insurance coverage. Connecticut and the Valdez-Cordova Census Area, Alaska, and its updated split FIPS codes (Chugach and Copper River Census Areas, Alaska) were excluded. Data not available for Kalawao County, Hawaii. Data for 2021 are available at https://www.census.gov/programs-surveys/sahie.html. ^†^ Percentage of households with a broadband Internet subscription. Valdez-Cordova Census Area, Alaska, and its updated split FIPS codes (Chugach and Copper River Census Areas, Alaska) were excluded. Five-year estimates (2018–2022) are available at https://www.census.gov/programs-surveys/acs. ^§^ Percentage of persons living in a household with income >100% of the federal poverty level. Valdez-Cordova Census Area, Alaska, and its updated split FIPS codes (Chugach and Copper River Census Areas, Alaska) were excluded. Data for 2022 are available at https://www.census.gov/programs-surveys/saipe.html. ^¶^ The county tertile cutoffs for the percentage of residents or households with a given factor were as follows: health insurance coverage: 53.7%–87.0%, 87.1%–91.7%, and 91.7%–97.6%; broadband Internet access: 36.0%–80.6%, 80.6%–86.0%, and 86.0%–100%; and income >100% of the federal poverty level: 57.6%–83.9%, 84.0%–88.3%, and 88.4%–96.9%. Percentages were rounded to one decimal place for readability, but groups do not overlap; statistical ranking was used to split counties into tertile groups before rounding. ** Rates were age-adjusted (calculated via direct method, using 2000 U.S. standard population) for race and ethnicity, sex, and urbanicity; used 10 categories for age adjustment: 0–4, 5–14, 15–24, 25–34, 35–44, 45–54, 55–64, 65–74, 75–84, and ≥85 years. Crude rates were used for age-stratified groups. ^††^ Hispanic or Latino (Hispanic) decedents could be of any race; all other racial and ethnic groups were non-Hispanic. ^§§^ Persons aged <10 years were not included in age-stratified rate ratios because of low death counts. ^¶¶ ^Rural-Urban Continuum Codes 1–3 were coded as urban, and Codes 4–9 were coded as rural. https://www.ers.usda.gov/data-products/rural-urban-continuum-codes/ *** Data from state vital records were used for 377 of 398 suicide deaths among Connecticut residents. ††† The x-axis is plotted on the log scale.

Among AI/AN persons, White persons, males, and adults aged 25–44 years, suicide rates were significantly lower among those who lived in counties in the highest and intermediate tertiles for health insurance coverage, broadband Internet access, and income than they were among persons who lived in counties in the lowest tertiles for these factors (Table 2). The magnitude of the RRs (i.e., rate in counties in the highest tertile compared with rate in counties in the lowest tertile) tended to be lowest (indicating that presence of the factor was most protective) in these groups and was particularly low for AI/AN persons, for whom the RRs ranged from 0.44 to 0.49 for counties in the highest versus the lowest factor tertiles (Figure 2). In other demographic groups, suicide rates were less consistently associated with these factors. For example, among females living in the lowest-income tertile counties, suicide rates were similar to those among females living in the highest-income tertile counties (RR = 0.98), and a similar pattern was observed among Black persons with respect to health insurance coverage (RR = 1.03). ^††††^

**TABLE 2:** Suicide rates by tertiles of selected county-level factors by demographic characteristics — National Vital Statistics System,* United States, 2022

Characteristic	Tertile^†^					
	Lowest		Intermediate		Highest	
	Deaths	Rate^§^	Deaths	Rate^§^	Deaths	Rate^§^
**Health insurance coverage**^¶,^**^,††,§§^						
**Race and ethnicity** ^¶¶^						
AI/AN	377	35.0	188	24.5***	85	15.4***
Asian and NH/PI	243	8.0	444	6.8***	851	7.0
Black or African American	1,151	9.0	1,393	8.7	1,246	9.2
White	9,855	22.2	11,809	18.6***	15,513	15.1***
Hispanic or Latino	1,979	9.0	1,777	7.7***	1,325	7.5***
Multiracial	139	10.2	191	9.8	348	11.2
**Sex** ^¶¶^						
Female	2,782	6.7	3,189	5.8***	4,135	5.6***
Male	10,984	26.5	12,667	23.3***	15,316	20.9***
**Age group, yrs** ^†††,§§§^						
10–24	1,874	11.6	2,172	10.2***	2,446	9.1***
25–44	4,768	22.1	5,537	19.0***	6,409	17.1***
45–64	4,211	21.2	4,883	18.8***	6,408	17.9***
≥65	2,911	20.6	3,260	18.5***	4,185	16.5***
**Urbanicity** ^¶¶,¶¶¶^						
Urban	10,396	15.3	12,947	13.5***	16,403	12.4***
Rural	3,370	21.1	2,909	20.1	3,048	18.8***
Broadband Internet access**^,^****						
**Race and ethnicity** ^¶¶^						
AI/AN	261	41.0	138	29.7***	251	19.3***
Asian and NH/PI	17	8.2	100	7.3	1,435	7.0
Black or African American	267	8.3	843	9.7***	2,711	8.8
White	3,371	22.7	8,009	19.8***	26,086	16.5***
Hispanic or Latino	296	9.5	824	8.9	3,999	7.9***
Multiracial	40	13.5	84	9.9	558	10.6
**Sex** ^¶¶^						
Female	758	7.2	1,905	6.3***	7,536	5.7***
Male	3,503	31.4	8,125	27.0***	27,623	21.3***
**Age group, yrs** ^†††,§§§^						
10–24	582	13.5	1,219	10.6***	4,725	9.6***
25–44	1,482	28.4	3,510	23.5***	11,846	17.2***
45–64	1,259	22.8	3,107	21.2***	11,273	18.1***
≥65	937	21.5	2,191	19.8***	7,310	17.3***
**Urbanicity** ^¶¶,¶¶¶^						
Urban	1,080	16.7	6,012	14.8***	33,001	13.0***
Rural	3,181	20.3	4,018	19.9	2,158	19.7
Income**^,§§,††††^						
**Race and ethnicity** ^¶¶^						
AI/AN	343	37.9	159	22.6***	148	18.5***
Asian and NH/PI	174	6.9	499	7.3	879	7.0
Black or African American	1,216	9.1	1,359	9.1	1,246	8.7
White	7,036	20.0	13,196	19.1***	17,234	15.8***
Hispanic or Latino	1,082	8.2	2,085	8.1	1,952	8.1
Multiracial	95	9.4	212	9.6	375	11.4
**Sex** ^¶¶^						
Female	1,949	5.9	3,544	6.0	4,706	5.8
Male	8,026	25.1	14,027	23.9***	17,198	21.4***
**Age group, yrs** ^†††,§§§^						
10–24	1,398	10.4	2,227	10.0	2,901	9.8
25–44	3,648	21.3	6,092	19.8***	7,098	17.2***
45–64	2,905	19.2	5,533	19.7	7,201	18.3***
≥65	2,020	18.6	3,716	18.7	4,702	17.4***
**Urbanicity** ^¶¶,¶¶¶^						
Urban	6,271	13.3	14,020	13.8***	19,802	13.1
Rural	3,704	20.5	3,551	20.1	2,102	18.9***

**Abbreviations:** AI/AN = American Indian or Alaska Native; FIPS = Federal Information Processing Standard; NH/PI = Native Hawaiian or Pacific Islander.

* Data from state vital records were used for 377 of 398 suicide deaths among Connecticut residents.

^†^ The county tertile cutoffs for the percentage of residents or households with a given factor were as follows: health insurance coverage: 53.7%–87.0%, 87.1%–91.7% and 91.7%–97.6%; broadband Internet access: 36.0%–80.6%, 80.6%–86.0% and 86.0%–100%; and income >100% of the federal poverty level: 57.6%–83.9%, 84.0%–88.3% and 88.4%–96.9%. Percentages were rounded to one decimal place for readability, but groups do not overlap; statistical ranking was used to split counties into tertile groups before rounding.

^§^ Suicide deaths per 100,000 population.

^¶^ Percentage of persons with health insurance coverage. Data for 2021 are available at https://www.census.gov/programs-surveys/sahie.html.

** Valdez-Cordova Census Area, Alaska, and its updated split FIPS codes (Chugach and Copper River Census Areas, Alaska) were excluded.

^††^ Connecticut was excluded.

^§§^ Data not available for Kalawao County, Hawaii.

^¶¶^ Age-adjusted rates (calculated via direct method, using 2000 U.S. standard population) used 10 categories for age adjustment: 0–4, 5–14, 15–24, 25–34, 35–44, 45–54, 55–64, 65–74, 75–84, and ≥85 years. Hispanic or Latino decedents could be of any race; all other racial and ethnic groups were non-Hispanic.

*** p<0.05 for difference with counties in the lowest tertile of factor based on Z-test for >100 deaths. When deaths were <100, differences in rates were considered significant if CIs based on a gamma distribution did not overlap.

^†††^ Crude rates.

^§§§^ Suppression of persons aged <10 years due to low death counts.

^¶¶¶^ Rural-Urban Continuum Codes 1–3 were coded as urban, and Codes 4–9 were coded as rural. https://www.ers.usda.gov/data-products/rural-urban-continuum-codes/

**** Percentage of households with a broadband Internet subscription. Five-year estimates (2018–2022) are available at https://www.census.gov/programs-surveys/acs.

^††††^ Percentage of persons living in a household with income >100% of the federal poverty level. Data for 2022 are available at https://www.census.gov/programs-surveys/saipe.html.

## Discussion

These findings highlight the importance of three county-level factors (health insurance coverage, household broadband Internet access, and household income) in relation to suicide rates. Overall, suicide rates in counties with higher levels of health insurance coverage, household broadband Internet access, and household income were lower than rates in counties with lower levels of these factors. There are several potential explanations for how these factors might protect against suicide. Health insurance might facilitate access to mental health services, as well as primary care and crisis intervention (*8*,*9*). Broadband Internet, recently referred to as a superdeterminant of health (*10*), can connect persons to job prospects, opportunities for social connectedness and support, and expanded access to medical services via telehealth (*7*,*10*). Living in higher-income communities is associated with ability to meet basic needs, such as food security and housing stability (*11*,*12*).

In addition, this analysis found that overall, higher suicide rates continue to affect certain sociodemographic groups, including rural residents, males, and AI/AN and White populations. For some sociodemographic groups included in the analyses, especially AI/AN persons, the three county-level factors examined might be particularly important. These findings are especially meaningful considering that some of these groups, such as AI/AN persons, are more likely to live in communities with lower levels of these factors, including broadband Internet access (*13*). The finding that higher levels of the three assessed factors are more strongly related to lower suicide rates among AI/AN persons and males aligns with previous studies examining economic factors (*14*,*15*). In contrast, the factors considered in this analysis were less clearly linked with suicide rates for some groups, such as Black persons. Other risk factors or protective factors not examined in this report might be more relevant among these populations. Additional community or societal factors, such as indicators of structural racism and stigma and norms around help-seeking, might influence the relationship between county-level factors and decreased suicide risk in certain populations (*16*,*17*). These findings highlight the need to examine risk and protective factors within populations and incorporate the findings of such research into suicide prevention practices.

A comprehensive approach to suicide prevention that targets both upstream and downstream prevention can promote these factors. This approach is laid out in the new 2024 National Strategy for Suicide Prevention (https://www.hhs.gov/nssp), which specifically highlights the importance of upstream prevention strategies. CDC’s Suicide Prevention Resource for Action (https://www.cdc.gov/suicide/resources/prevention.html) aligns with the National Strategy and describes policies, programs, and practices with the best available evidence that states and territories, tribes, and communities can implement to address suicide risk and protective factors at the individual, relationship, community, and societal levels (*3*). Relevant upstream strategies include strengthening economic supports (e.g., strengthening household financial security, such as through the Supplemental Nutrition Assistance Program and stabilizing housing), improving access and delivery of suicide care (e.g., Zero Suicide^§§§§^), promoting healthy connections (e.g., community engagement), teaching coping and problem-solving skills, and creating protective environments (e.g., creating healthy organizational policies and culture). These strategies are being implemented in populations disproportionately affected by suicide through CDC’s Comprehensive Suicide Prevention Program (CSP) (https://www.cdc.gov/suicide/programs/csp.html). For example, in addition to conducting a public health campaign to reduce stigma and training providers in hospital and emergency departments on suicide prevention approaches, the CSP recipient in Vermont is specifically supporting rural populations, including farmers, through peer support networks and increasing providers’ abilities to reach and deliver tele-mental health to these populations using telehealth. The CSP recipient in Colorado is not only working with counties and local organizations to promote connectedness for populations at high risk for suicide and providing gatekeeper trainings to help identify and connect persons at risk for suicide with the support services they need but is also working to strengthen community factors that protect against suicide by developing partnerships to support economic stability initiatives, such as food security, affordable housing, and transportation (https://www.cdc.gov/suicide/csp-profiles/index.html).

### Limitations

The findings in this report are subject to at least five limitations. First, although these findings highlight associations between health insurance coverage, household broadband Internet access, household income, and decreased suicide rates, this study had an ecologic design and thus did not make causal inferences. The possibility of confounding other than by demographic factors was not addressed. Second, it was not possible to examine some disproportionately affected populations, including veterans, persons with disabilities, and sexual and gender minorities (*2*). Third, factors were measured at the county level; smaller geographic units (e.g., official U.S. census tracts) might better represent communities and be more closely associated with reduced suicide risk (*18*). Fourth, rates by race and ethnicity could reflect underreporting of deaths in the vital statistics data, particularly for AI/AN and Hispanic persons, thereby underestimating rates in these populations (*19*,*20*). Finally, other county-level factors that might be relevant to suicide prevention were not examined in this analysis.

### Implications for Public Health Practice

Improving the conditions where persons are born, grow, work, live, and age might reduce suicide deaths (*4*). Decision-makers, government agencies, and communities can work together to implement programs, practices, and policies that increase access to health insurance and broadband Internet and promote economic supports; this approach is especially important for populations disproportionately affected by suicide. Combined with downstream actions that support persons at increased or immediate risk for suicide (e.g., crisis care or the 988 Suicide & Crisis Lifeline; https://www.988lifeline.org), an upstream approach that promotes these factors might be an important component of suicide prevention. More attention to such upstream strategies that prevent suicide crises before they start has the potential to accelerate public health’s ability to save lives. 

## Footnotes

*
https://www.cdc.gov/about/priorities/why-is-addressing-sdoh-important.html

†
https://www.cdc.gov/nchs/nvss/deaths.htm

§ To incorporate data from all 50 states, vital records from Connecticut supplemented NVSS files. This strategy was necessary for analyses that incorporated county-level measures because 2022 NVSS county information is classified based on Connecticut’s eight counties, but all U.S. Census Bureau products from 2022 forward only contain Connecticut’s nine planning regions as county-equivalents. To fill this gap, Connecticut vital statistics provided data for persons who died by suicide in Connecticut, representing 377 of 398 suicide deaths among Connecticut residents.

¶ Asian and NH/PI were combined because the number of deaths for NH/PI alone would have yielded suppressed rates.

** Suicide deaths among persons aged <10 years were suppressed because of low death counts.

†† The U.S. Department of Agriculture urbanicity scheme was used because it is the most current urbanicity scheme. Rural-Urban Continuum Codes 1–3 were coded as urban, and Codes 4–9 were coded as rural. https://www.ers.usda.gov/data-products/rural-urban-continuum-codes

§§ SAHIE measures any type of health insurance coverage. SAHIE estimates reflect county estimates of health insurance coverage among persons aged <65 years because health insurance coverage among persons aged ≥65 years is nearly universal. All ages were included in analyses of overall rates and by race and ethnicity, sex, and urbanicity because subanalyses of the ≥65 years age group demonstrated associations between county-level health insurance coverage and suicide rates. https://www.census.gov/programs-surveys/sahie.html

¶¶
https://www.census.gov/programs-surveys/acs

***
https://www.census.gov/programs-surveys/saipe.html

††† The county tertile cutoffs for the percentage of residents or households with a given factor were as follows: health insurance coverage: 53.7%–87.0%, 87.1%–91.7% and 91.7%–97.6%; broadband Internet access: 36.0%–80.6%, 80.6%–86.0% and 86.0%–100%; and income >100% of the federal poverty level: 57.6%–83.9%, 84.0%–88.3% and 88.4%–96.9%. Percentages were rounded to one decimal place for readability, but groups do not overlap; statistical ranking was used to split counties into tertile groups before rounding.

§§§
https://wonder.cdc.gov/wonder/help/ucd-expanded.html#Age%20Adjustment

¶¶¶ 45 C.F.R. part 46, 21 C.F.R. part 56; 42 U.S.C. Sect. 241(d); 5 U.S.C. Sect. 552a; 44 U.S.C. Sect. 3501 et seq.

**** Percent reduction as calculated by the formula: ([rate for highest tertile of factor – rate for tertile level of factor] / rate for highest tertile of factor) × 100. Percent reduction was calculated using exact, unrounded rates.

†††† RRs were calculated using exact, unrounded rates.
